# Optimized silk fibroin piezoresistive nanocomposites for pressure sensing applications based on natural polymers

**DOI:** 10.1039/c8na00417j

**Published:** 2019-04-22

**Authors:** Ander Reizabal, Sérgio Gonçalves, Ricardo Brito-Pereira, Pedro Costa, Carlos M. Costa, Leyre Pérez-Álvarez, Jose Luis Vilas-Vilela, Senentxu Lanceros-Méndez

**Affiliations:** BCMaterials, Basque Center for Materials, Applications and Nanostructures, UPV/EHU Science Park 48940 Leioa Spain senentxu.lanceros@bcmaterials.net; Macromolecular Chemistry Research Group (LABQUIMAC), Dept. of Physical Chemistry, Faculty of Science and Technology, University of the Basque Country (UPV/EHU) Spain; Center of Physics, University of Minho 4710-058 Braga Portugal cmscosta@fisica.uminho.pt; Centro ALGORITMI, University of Minho, Campus de Azurém 4800-058 Guimarães Portugal; EngageLab, University of Minho 4810-453 Guimarães Portugal; Institute for Polymers and Composites IPC/i3N, University of Minho 4800-058 Guimarães Portugal; Center of Chemistry, University of Minho 4710-058 Braga Portugal; Ikerbasque, Basque Foundation for Science 48013 Bilbao Spain

## Abstract

Environmental issues promote the development of sensors based on natural polymers which are becoming an area of increasing interest. Piezoresistive sensors based on silk fibroin with carbon nanotubes (CNTs) as fillers were produced by solvent-casting in order to tune their electrical conductivity and electromechanical responses. It is shown that the carbonaceous fillers are well dispersed in the polymer matrix and the thermal and mechanical properties are independent of the CNT content. On the other hand, the inclusion of CNTs reduces the β-sheet content of silk fibroin and the electrical properties of the composite strongly depend on the filler content, the percolation threshold being around 1 wt% CNTs. The piezoresistive response demonstrates good reproducibility during cyclic loading without hysteresis with a piezoresistive sensitivity of ∼4 MPa^−1^, regardless of the CNT content. Overall, the results confirm that polymer composites based on natural polymers exhibit excellent piezoresistive responses, also demonstrated by the implementation and testing of a pressure sensor with the corresponding readout electronics. Thus, it is shown that natural polymers such as silk fibroin will allow the development of a new generation of multifunctional force and deformation sensors.

## Abstract 2

Environmental issues promote the development of sensors based on natural polymers which are becoming an area of increasing interest.

## Graphical abstract

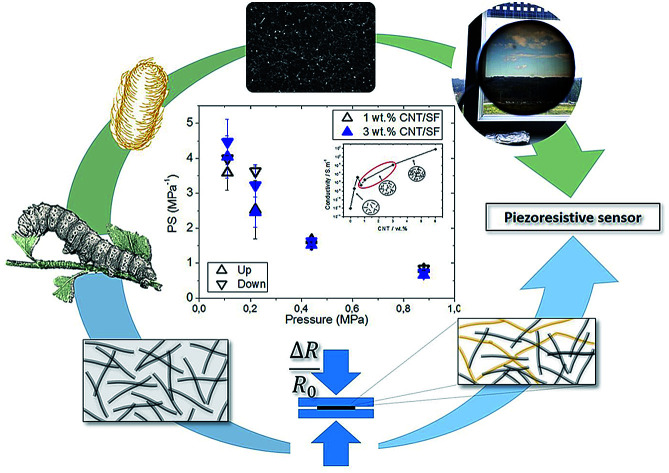

## Introduction

Remarkable achievements have been obtained with polymer composites allowing the improvement of their thermal, mechanical and electrical properties for application in areas such as construction, electronics, consumer products, sensors and actuators, biological applications, and energy, among others.^1–3^

The main recent focus is to obtain high performance composite materials based on natural polymers and biopolymers, in order to reduce the use of polymers obtained from crude oil, while maintaining functional performance.^4^ The prefix ‘bio’ in biopolymers means that they are produced from biological sources, including biomass – *e.g.* agro-polymers – as well as by microbial production or other living organisms.^5,6^ Starches, pectins, chitosan/chitin, collagen/gelatin, poly(hydroxybutyrate) (PHB), poly(lactic acid) (PLA), or silk fibroin (SF) are some of the more reported biopolymers in the literature.^5^

In particular, silk fibroin, mainly obtained from the *Bombyx mori* cocoon, deserves special attention among natural polymers due to its non-toxicity, excellent mechanical, thermal and electrical properties (*e.g.* piezoelectric response), biocompatibility and controllable biodegradability, being obtained in a wide range of morphologies and applied in a wide range of applications ranging from textile to healthcare sectors.^7,8^

Silk fibroin is composed of glycine (45%), alanine (30%), and serine (12%) in a roughly 3 : 2 : 1 ratio and dominated by [Gly-Ala-Gly-Ala-Gly-Serl]_*n*_ sequences with β-sheet crystallites and amorphous domains.^9,10^

A wide variety of structures and morphologies can be obtained from silk fibroin,^11^ its functional characteristics and processability supporting the selection of silk fibroin^12^ for a wide range of applications in the areas of biomedicine,^13^ opticals, photonics and electronics.^14,15^

Piezoresistive sensors based on natural polymers are useful for force and deformation measurements, and have a high potential in the scope of Industry 4.0 and Internet of Things paradigms, which increasingly demand a large variety of environmentally friendlier sensors.^16,17^

There are different types of piezoresistive sensors; however, taking into account their flexibility, easy processability and integrability, the most outstanding are piezoresistive polymer based composites.^18,19^ These composites are composed of a polymer matrix and conductive fillers, the most used ones being carbonaceous fillers such as carbon black,^20^ carbon nanofibers (CNFs),^21^ carbon nanotubes (CNTs)^22^ and graphene.^23^ The final properties of the composites (electrical, mechanical, thermal and electromechanical) are determined by filler type, content and aspect ratio.^24^ Polymer matrices typically used for piezoresistive composites include thermoplastics^25^ and elastomers^26^ but the use of natural polymers is scarce in the literature.

Some studies have been reported on polymer composites based on silk fibroin and carbon nanotubes (CNTs) for biomedical applications^27,28^ and enzymatic biofuel cells.^29^ The silk fibroin/CNT composites were obtained by the template method leading to a conductive composite that was used as a permissive neural interface allowing adhesion and differentiation of dorsal root ganglion neuronal cells (DRG) *in vitro*.^27^

In addition, electrospinning has been used to prepare silk fibroin/CNT composites using formic acid as a solvent. It is observed that carbon nanotubes are incorporated along the nanofibers, leading to enhanced mechanical properties with the incorporation of small amount of CNTs.^28^

Further, silk fibroin cryogels with carbon nanotubes were fabricated by a sol–gel process followed by freeze-drying in which the carbon nanotubes were incorporated within the porous three-dimensional silk fibroin network structure, leading to a change in the crystal structure of silk fibroin.^30^

In order to explore and expand the applicability of silk fibroin, this work proposes silk fibroin nanocomposites based on carbon nanotubes for the fabrication of piezoresistive sensors. The aim of this work is to evaluate the morphological, thermal, mechanical and electrical properties of silk fibroin films with different amounts of carbon nanotubes, paying particular attention to the electrical and electromechanical properties in order to develop multifunctional materials for sensor and actuator applications.

## Experimental

### Materials

*Bombyx mori* cocoons were supplied by APPACDM from Castelo Branco (Portugal). Carbon nanotubes (CNTs) were provided by Nanocyl (reference NC7000, purity of 90%, 1.5 μm length and an outer mean diameter of 9.5 nm).

### Composite film preparation

*Bombyx mori* cocoons were cut into small pieces and degummed by boiling them for 30 min in a 0.02 M Na_2_CO_3_ solution. For each 1 g of silk, 40 ml of aqueous Na_2_CO_3_ solution were used. After that, the fibers were rinsed several times with distilled water, squeezed to remove excess water, and finally dried overnight in a fume hood. Once the fibers were totally dried, they were dissolved in a 0.2 M formic acid/CaCl_2_ (FA) solution with 10 ml FA per gram of SF. After centrifugation to remove insoluble residues, the silk solution was placed in an airing chamber to allow fast evaporation of formic acid within 24 h. CaCl_2_ was removed by progressive washing with distilled water until the solution showed a constant conductivity. Finally, purified SF was dried overnight in a fume hood.

**Fig. 1:**
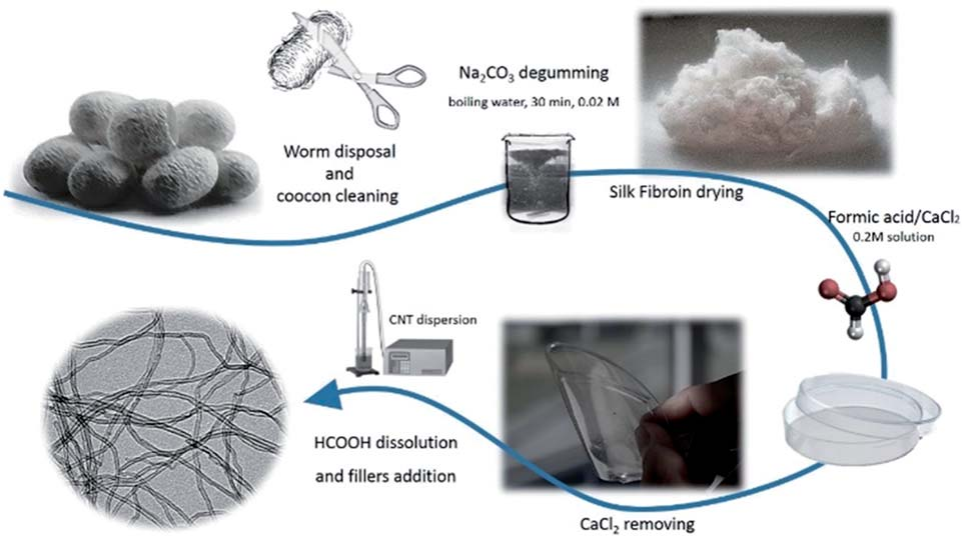
Main experimental steps for the processing of SF/CNT composites.

For SF/CNT composite preparation, solutions were prepared in formic acid with different amounts of CNTs (0.25, 0.5, 0.75, 1, 3, and 6 wt%, with respect to SF). Fillers were initially added to formic acid and mixed in an ultrasonic bath for 3 h to ensure good dispersion and to prevent nanotube agglomeration. Afterwards, SF (8 wt%) was added. Once proteins were completely dissolved under magnetic stirring, the solution was sonicated using a sonication needle for 5 minutes with 30% of amplitude, in cycles of 7 seconds of sonication and 3 seconds of landing to ensure the correct dispersion of the fillers. The obtained SF/CNT mixture was cast overnight in polypropylene Petri dishes and dried to remove all the formic acid and promote β-sheet crystal formation. The processing steps and corresponding sequence are illustrated in Fig. 1.

### Characterization techniques

The morphology of the samples and the dispersion of CNTs were examined using a scanning electron microscope (SEM, NanoSEM – FEI Nova 200 (FEG/SEM)) with an accelerating voltage of 15 kV. The films were previously deposited with a conductive gold layer by sputtering with Polaron SC502 apparatus.

The structure of the silk fibroin films and possible interaction between the CNTs and the polymer matrix were evaluated by Fourier Transform Infrared Spectroscopy (FTIR) performed at room temperature with a Jasco FT/IR-4100 system. FTIR spectra were collected in ATR mode from 4000 to 600 cm^−1^ after 64 scans with a resolution of 4 cm^−1^.

In order to determine the relative content of the secondary structures present in each sample, the deconvolution of the band in the spectral region corresponding to amide I was carried out with OriginPro 8.1 software (OriginLab, Northampton).

The thermal behavior of the samples was determined by Differential Scanning Calorimetry (DSC) and Thermogravimetric Analysis (TGA).

DSC was performed with Mettler Toledo DSC 822e apparatus with a sample robot between 25 and 350 °C at 10 °C min^−1^ under nitrogen purge (50 mL min^−1^) in 40 μL aluminium cans with perforated lids. TGA was carried out using a TGA/SDTA 851e Mettler Toledo apparatus under a flow rate of 50 mL min^−1^ operating between 25 and 800 °C at 10 °C min^−1^.

Dynamic mechanical analysis (DMA) was performed with Mettler Toledo DMA1 apparatus in tensile mode. The storage modulus and loss tangent were measured as a function of temperature at a frequency of 1 Hz from 0 °C to 280 °C and a heating rate of 3 °C min^−1^. The measurements were performed on samples with typical dimensions of 10 × 4 × 0.050 mm.

Dielectric measurements were performed using a Quadtech 1920 LCR precision meter. The capacity and the dielectric losses (tan *δ*) were obtained at room temperature in the frequency range of 20 Hz to 1 MHz with an applied voltage of 0.5 V. Circular gold electrodes of 5 mm diameter were deposited on both sides of each sample with a Polaron SC502 sputter system. The error associated with the dielectric measurements is ∼1%, mainly due to the determination of the geometrical parameters with a Mitutoyo micrometer.

The real part of the dielectric function (*ε*′) and the a.c. electrical conductivity (*σ*′) were determined using the following equations:1
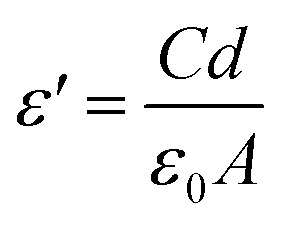
and

2 *σ*′ = *ε*_0_*ωε*′ tan *δ*

where *C* is the capacitance (F), *ε*_0_ is the permittivity of vacuum (8.85 × 10^−12^ F m^−1^), *A* is the electrode area (m^2^), *d* is the thickness of samples (m) and *ω* = 2π*ν* is the angular frequency.

The d.c. electrical conductivity of the samples was obtained by a 2-wire method with an applied voltage between ±10 V and measuring the current using a Keithley 487 picoammeter/voltage source. The volume resistivity was measured at room temperature in samples with circular contacts of 5 mm diameter and the resistivity of the samples (*ρ* in S m^−1^) was obtained from the slope of the *I*–*V* curves and calculated using:3
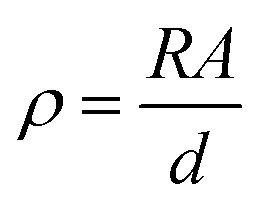
where *R* is the resistance of the sample, *d* is its thickness and *A* is the electrode area.

Electro-mechanical tests were performed by measuring the volume electrical resistance (Agilent 34401A multimeter) in real time using the two-probe method while the samples were subjected to a cyclic compression load using a Shimadzu AG-IS with a load cell of 500 N (Instron 5544) at 2 mm min^−1^.

The electro-mechanical test results under compression were used to evaluate the pressure sensitivity (PS) using the following equation:^31^4
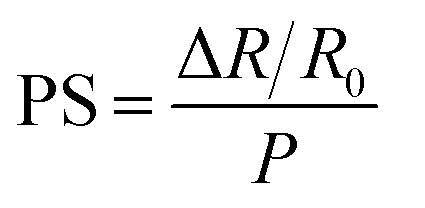
where *R*_0_ is the initial electrical resistance in the unloaded state, Δ*R* is the electrical resistance change and *P* is the applied pressure.

For each experimental technique at least three measurements were performed for each sample.

## Results and discussion

### Morphological and FTIR characterisation

**Fig. 2:**
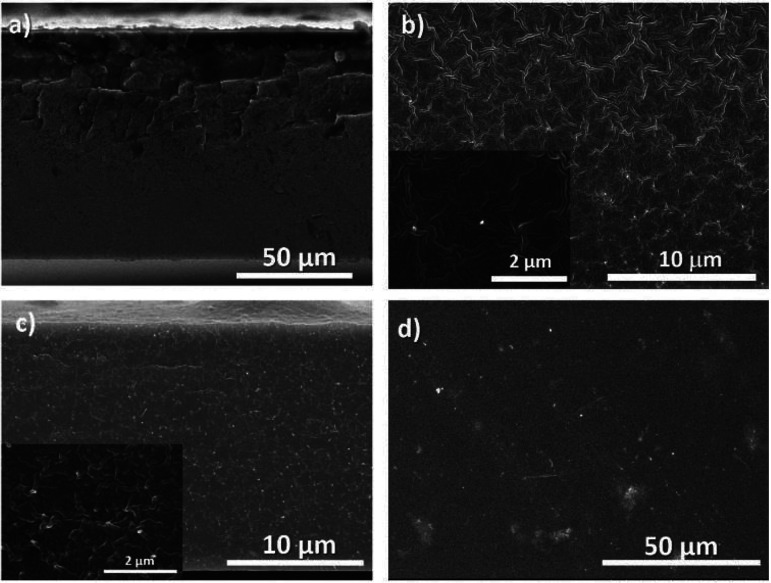
SEM cross-sectional images of neat SF (a) and SF/CNT composites with 0.75 wt% CNTs (b) and 6 wt% CNTs (c) and the surface image of 6 wt% CNTs (d).

The morphology of the samples and the dispersion of the CNTs were evaluated by SEM, as shown in Fig. 2. Fig. 2 shows the SEM images of SF/CNT composites with 0, 0.75 and 6 wt% CNT content, and these images were representative of the rest of the concentrations.

It is shown that the compact morphology of neat SF (Fig. 2a) is preserved in the composites (Fig. 2b and c). A good dispersion of CNTs (bright spots in Fig. 2b and c) is observed for all the composites, independent of the filler content. Larger agglomerates were observed for the SF/CNT composites, with CNT clusters of ∼1–2 μm in size, well distributed throughout the sample.

Fig. 2c and d (6 wt% CNT composite) show that the fillers are also uniformly distributed along the cross-section of the samples, allowing suitable mechanical and electrical macroscopic responses (see later). The inset in Fig. 2c also shows the proper wetting of the CNTs by the polymer.

As observed by Zhou *et al.*, SF films exhibit a globule-like nanostructure.^32^ Films regenerated from formic acid solvent casting show a globule-like size of around 100 nm^32^ as a consequence of SF chains self-assembling during the solvent evaporation process. SF/CNT composites show a similar structure for the different CNT concentrations.

**Fig. 3:**
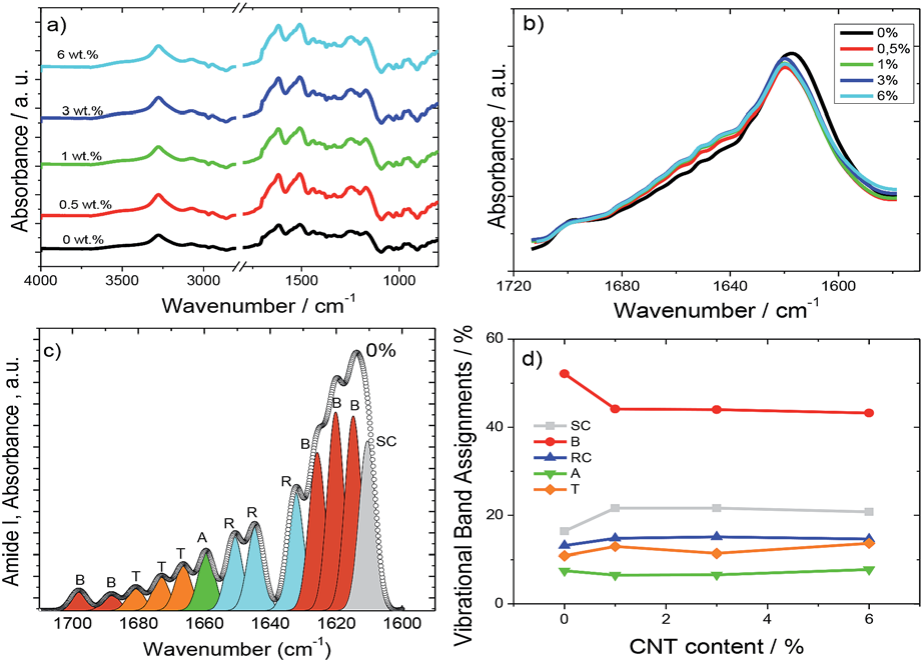
(a) Full-range ATR-FTIR spectra for neat SF and the different composites. (b) Amide I region of the ATR-FTIR spectra for all SF/CNT composites. (c) Typical deconvolution spectrum of amide I for neat SF and (d) integral area fraction of the different spectral components resolved in this spectral region. The different contributions to the amide I envelope are marked as random coils (RC, blue), β-sheets (B, red), α-helices (A, green), turns (T, orange), and side chains (SC, grey).

In order to gain insight into the secondary structural changes of SF chains with the addition of the fillers, the ATR-FTIR spectra of the composites are presented in Fig. 3. Fig. 3 shows the full-range ATR-FTIR spectra in which the frequency range from 1800 cm^−1^ to 1400 cm^−1^ contains the most important amide I and amide II regions, describing the metastable state between a partially ordered α-helix and the β-sheet structures and the antiparallel β-sheet structure, respectively.^33,34^

The strong absorption at 3500–3000 cm^−1^ in Fig. 3a represents the OH stretching vibration, H-bond and NH stretching vibration.^35^

The secondary structures of SF are usually determined after the amide I band between 1700 and 1600 cm^−1^ (Fig. 3b) associated with the C=O stretching vibration, NH in plane bending, out of phase CN stretching vibration, and CCN deformation.^36^Fig. 3b shows that the inclusion of CNTs affects the vibration bands of SF but that the effect is independent of CNT content.

Amide I signals correspond to the vibration of different secondary protein structures such as side chains (1605–1615 cm^−1^), β-sheets (1619–1628 and 1697–1703 cm^−1^), random coils (1638–1655 cm^−1^), α-helices (1656–1662 cm^−1^), and turns (1663–1696 cm^−1^).^37–39^ Thus, they can be differentiated, as shown in Fig. 3c, in a typical deconvolution indicating the corresponding area for each component for pure SF. The same procedure was performed for all SF/CNT composites and the results are summarized in Fig. 3d.

Fig. 3d shows that the dominant conformation of SF is the β-sheet with 52%. This conformation is reduced to 44% for the SF/CNT composites independent of CNT content.^36^ This can be ascribed to a reduction in the degree of crystallinity of the samples,^36^ which is mainly attributed to the defects induced by the nanofillers as well as to noncovalent interactions between nanocarbon materials and silk fibroin through physisorption.^40^

Fig. 3d reveals that the proportion of the random coils and turns is practically the same (15 *vs.* 13%, respectively) and the α-helix conformation is approximately 7% for all SF/CNT composites. Thus, the presence of the fillers does not modify the SF conformations.

### Thermal and mechanical characterisation

The thermal and mechanical characterization of SF/CNT composites was performed to evaluate their dependence on CNT content, due to the thermal conductive properties and mechanical reinforcement effect of CNTs.^41^

**Fig. 4:**
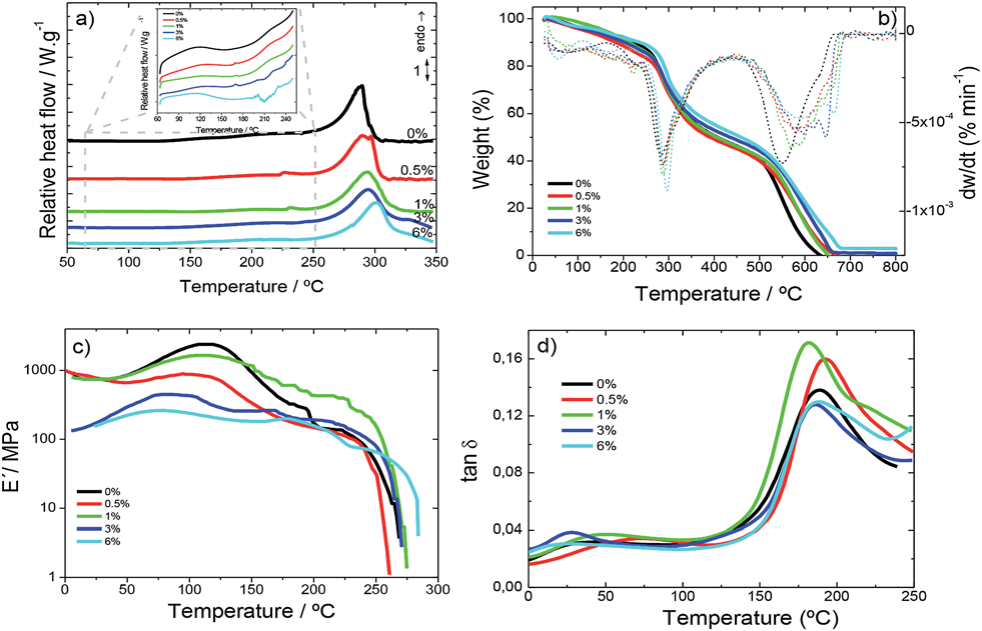
(a) DSC curves in the heating scan; (b) TGA thermograms; (c) viscoelastic modulus (*E*′) and (d) loss tangent (tan *δ*) for SF and the corresponding SF/CNT composites.

DSC thermograms of the SF/CNT composites shown in Fig. 4a demonstrate that all samples exhibit a similar thermal behaviour characterized by an endothermic peak around 290 °C associated with the degradation of SF molecular chains. Melting endothermic peaks were not observed in any samples because silk fibroin degrades at temperatures lower than the melting point.

During the heating scan an endothermic peak at 100 °C attributable to evaporation of bonded and non-bonded water was observed (Fig. 4a).^42^ Some signals ascribed to the loss of water show the ability of the samples to absorb ambient water during the casting process.

During the DSC heating scans, no relevant variations in the glass transition or random coil/α-helix to β sheet conformational changes were observed.^43^ This behaviour usually observed in highly crystalline samples corroborates the FTIR data. The thermal degradation endothermic peak was not influenced by filler addition; thus CNT–SF interactions do not influence chain packing and degradation.

TGA thermograms are essential for the determination of the temperature range in which the samples will not undergo any weight loss process affecting the macroscopic response of the materials. Further, they enable the evaluation of water content in the samples since SF has the ability to contain water molecules as shown in Fig. 4b.^42^

Fig. 4b shows the TGA thermogram for all SF and SF/CNT samples, demonstrating that the decomposition of the samples occurs in two main stages.

An initial weight loss is observed up to 150 °C for all samples (Fig. 4b) due to water removal (evaporation), corresponding with the first DTGA peak. The storage of water in silk films can be of two different types: free water molecules that can be easily removed and, therefore, be the first to evaporate on heating and bonded-water, bonded to protein chains through interactions between the amide groups of proteins and the OH groups of water.^42^

The water content of the silk samples was determined from the weight decrease in the first step (Fig. 4b) and a similar water content of ∼10% is seen in all the samples, since CNTs do not alter the ability of silk fibroin to stock water.

The second degradation step ranges from 200 °C to 270 °C and corresponds to the degradation of the samples through the breakage of the primary chain of the silk molecules. This step is independent of the water content^42^ and the inclusion of the CNTs.

Before the second degradation peak in the DTGA curve (Fig. 4b) a small peak at 235 °C is observed corresponding to the degradation of the less stable crystal domains not observed in pure SF samples.

The mechanical properties of the films, *i.e.*, the elastic modulus (*E*′) and the loss tangent (tan *δ*), are shown as a function of temperature (0–280 °C) at 1 Hz in Fig. 4c and d.

For all SF and SF/CNT composites (Fig. 4c) the elastic modulus remains constant until 50 °C, sharply increases at about 100 °C and then decreases abruptly at about 150 °C. This behaviour predicts the water molecule plasticising effect over SF and results in the increase of the storage modulus as a consequence of evaporation. With increasing temperatures above 150 °C, the storage modulus decreases until 280 °C where the modulus strongly drops as a consequence of the beginning of polymeric structure degradation, as shown in the TGA thermograms (Fig. 4b).

The loss tangent (Fig. 4d) increases slowly up to about 150 °C and increases sharply above this temperature until it reaches a peak at about 180 °C.

The decrease in the elastic modulus (Fig. 4c) at about 150 °C and the peak of the loss tangent (Fig. 4d) at 200 °C are explained in terms of the segmental motion of the main chains of the silk fibroin molecules in the amorphous film.^44^

The storage modulus of the SF/CNT composites decreases with increasing CNT content. Thus, the CNTs act as defects in the SF structure and, therefore, deteriorate the mechanical properties of the composites. This fact is particularly relevant for the SF/CNT composites with 6 wt% filler content in which the storage modulus decreases by two orders of magnitude when compared to the samples with the lowest CNT content (Fig. 4c).

The CNT contents of 0.5 and 1 wt% in SF/CNT composites are not enough to produce significant mechanical variation and these composites show a similar behaviour to neat SF.

In addition, a slight destabilization of the glass transition temperature (*T*_g_) is observed as indicated by the shift of the peak of tan *δ* (Fig. 4d) around 170–180 °C to lower temperatures with increasing carbon nanotube content.

### Electrical and electro-mechanical behaviour

**Fig. 5:**
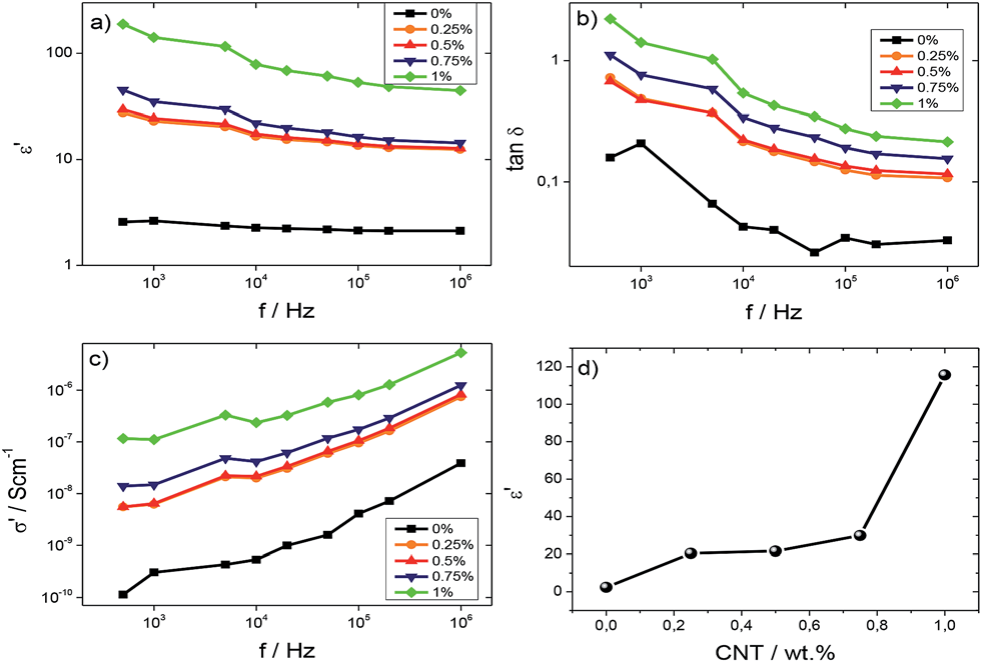
(a) Dielectric constant, *ε*′, (b) tan *δ* and (c) a.c. conductivity for all SF/CNT composites. (d) Variation of the dielectric constant as a function of CNT content at 5 kHz.

For sensor applications, the electrical properties can be tuned by the inclusion of CNTs. The dielectric response (*ε*′, tan *δ* and *σ*′) calculated using eqn (1) and (2) for the different samples is shown in Fig. 5.

Fig. 5a and b show the dielectric constant and loss tangent, respectively, for SF and SF/CNT composites, except for the composite with 6 wt%, which is very conductive, as a function of the frequency at room temperature. For all samples, Fig. 5 a and b show that the dielectric constant and tan *δ* decrease as the frequency increases due to slow dipole mobility.^45^ It is also detected that the dielectric constant increases with the addition of the CNTs, regardless of the frequency, due to increased charge carriers, with eventual contributions from the Maxwell–Wagner–Sillars (MWS) effect due to interfacial charge accumulation under an external electric field (Fig. 5a).^46^ This is also confirmed by the increase of both tan *δ* and *σ*′ as shown in Fig. 5b and c.

**Fig. 6:**
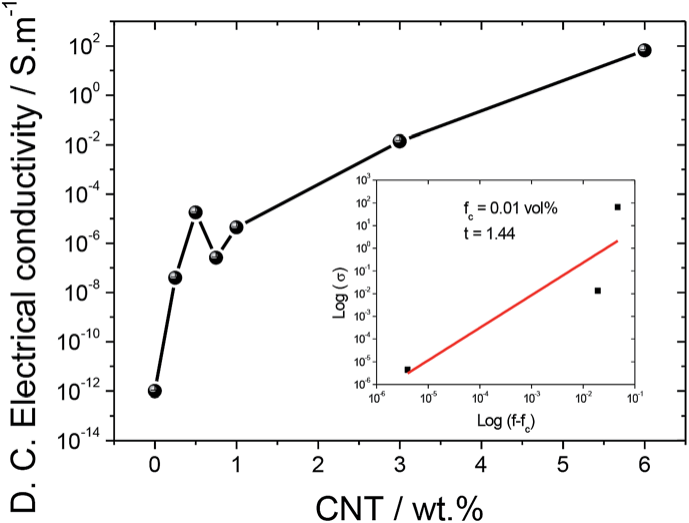
d.c. electrical conductivity of SF and the SF/CNT composites.

Fig. 5c shows the *σ*′ values for the different samples as a function of frequency. It is verified that the electrical conductivity increases with increasing frequency at room temperature, independent of the CNT content, indicating increased charge carrier mobility at localized states. The a.c conductivity increases with addition of CNTs due to both the introduction of additional charge carriers and the formation of a conductive network, until reaching a percolation, as shown in Fig. 6 for the d.c. conductivity.^47–49^

Fig. 5d shows the room temperature dielectric constant of the samples at 5 kHz as a function of CNT content. The dielectric constant increases with increasing CNT content due to contributions of the localized charge movement and the formation of local microcapacitors, as explained by the percolation theory.^50^

In order to determine the percolation threshold, the d.c. electrical conductivity was evaluated and the results as a function of CNT content are shown in Fig. 6.

The d.c. electrical conductivity increased by over 14 orders of magnitude when the filler content increased up to 6 wt%, the percolation threshold being at approximately 1 wt% CNTs.

The results in Fig. 6 show that the carbon nanotube conductive network is formed within the silk fibroin polymer. The electrical conductivity of SF/CNT composites can be theoretically predicted by the percolation model through the following equation:^51^

5 *σ* ≈ (*f* − *f*_c_)^*t*^ for *f* > *f*_c_

where *f* and *f*_c_ are the volume fraction of CNTs and the percolation threshold of the SF/CNT composites, respectively, and *t* is the critical exponent in the conducting region.

It can be seen in the inset of Fig. 6, after the application of eqn (5), that the percolation volume is *f*_c_ = 0.01 vol% (1 wt%) and the critical exponent in the conducting region is *t* = 1.44. It is notable that the critical exponent obtained for SF/CNT composites is 1.44, close to 1.1–1.3 for two-dimensional systems.^52^

Taking into account the electrical results in Fig. 6, the electromechanical performance was evaluated for SF/CNT composites with 1 and 3% wt% CNTs, as shown in Fig. 6. It has been concluded that the piezoresistive response of carbonaceous composites is the largest around the percolation threshold due to larger variations in the conductive network under mechanical solicitation.^22^

**Fig. 7:**
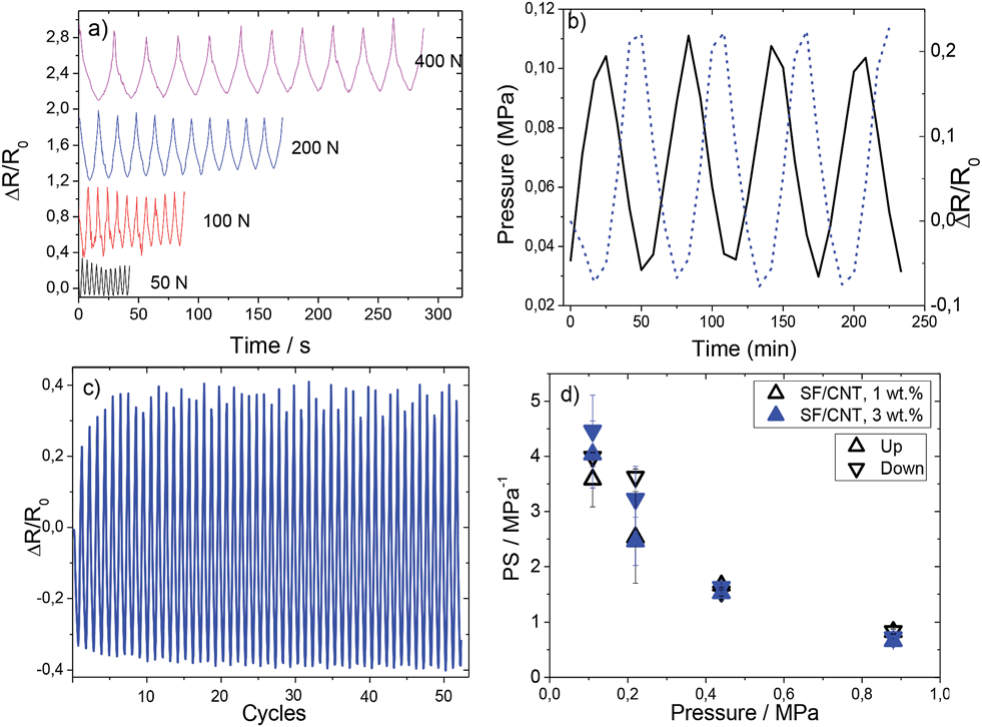
(a) Mechanical behaviour of the SF/CNT composites with several applied forces (from 50 to 400 N). (b) Pressure and relative resistance variation as a function of time (up to 50 N or 0.11 MPa) for 1 wt% CNTs and (c) stability over repeated cycling (more than 50 cycles) for the same sample. (d) Piezoresistive performance in pressure sensibility (PS) up to pressures near 1 MPa for SF/CNT composites with 1 and 3 wt% CNTs.

The electromechanical response was evaluated under compressive loading cycles with a maximum applied pressure of 400 N (equivalent to 0.88 MPa), as shown in Fig. 7.

The loading–unloading pressure cycles from 50 up to 400 N are presented in Fig. 7a, for 10 cycles in each measurement. Fig. 7b shows the relative variation of resistance during cycle loading and pressure application variation at a strain rate of 1 mm min^−1^ for SF/CNT composites with 1 wt% CNTs. This behavior is similar to that of the sample with 3 wt% filler content.

It is observed that the piezoresistive behaviour presents an excellent response with a small hysteresis effect (Fig. 7b and c). The stability of the cyclic response is shown in Fig. 7c by the resistance variation for 50 cycles. The minimum and maximum electrical resistance is constant with cycles (except for the initial 6–8 cycles); thus, we can conclude that the mechanical stability of these composites is good and they can be used as sensor materials.

It can be seen that SF/CNT composites present good reproducibility and signal stability and that the conductive network variations of carbon nanotubes in silk fibroin is stable, the resistance variations upon mechanical cycling being explained by variations of the tunnelling distance among fillers.^53^ This fact is verified for both composites (1 and 3 wt%).

On applying varying maximum pressures (Fig. 7d) a decrease of the piezoresistive sensitivity (PS) with increasing pressure can be observed: the application of increasing pressure leads to permanent reorientation and reconfiguration of the fillers and, eventually, conductive network breakage.^31^ It is observed that the PS value is independent of CNT amount and it is the lowest (0.72 MPa^−1^) for 0.88 MPa, due to the aforementioned permanent deformation of the conductive network.

The piezoresistive sensor based on silk fibroin shows an excellent piezoresistive sensitivity value and represents an advance in comparison with the ones shown in the state-of-the-art for conventional polymers (*i.e.*, thermoplastic polyurethane (TPU),^54,55^ polyester-based TPU,^56^ silicone rubber^57^ and polydimethylsiloxane (PDMS)^58^) in terms of environmental friendliness.

### Implementation of a pressure sensor prototype based on SF/CNT composites

As a proof of concept of the suitability of the materials for sensor applications, a simple prototype was fabricated envisioning that it could be used as a touch/pressure sensor, for example, by being embedded inside the paper sheets of a hybrid book, allowing digital interaction with the user, but without the negative environmental impact that plastic-based sensors usually have. Therefore, the developed silk-based material can be used as a touch/pressure sensor, either analogically or digitally.

**Fig. 8:**
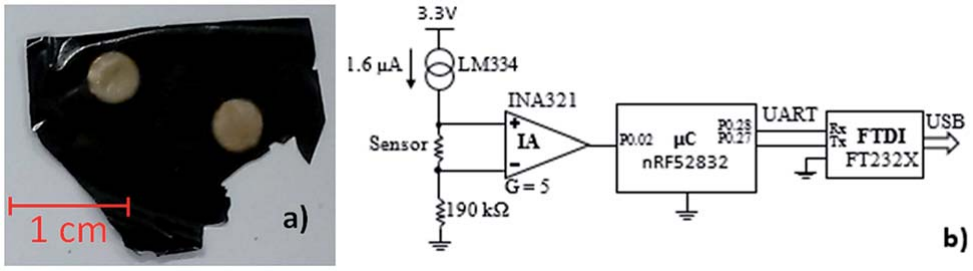
(a) SF/CNT 1 wt% CNT content film with 4 gold electrodes (2 on the top and 2 on the bottom); (b) schematic of the test circuit, consisting in a current source and an instrumentation amplifier (IA).

To achieve this goal, two pairs of gold electrodes were deposited on the film using the sputtering technique, two on the top layer and the other two on the bottom layer, so that each pair was overlapping, as presented in Fig. 8a. A SF/CNT film with 1 wt% CNT content was used due to its electrical resistivity: ≫ 200 MΩ between side electrodes, and 150 to 300 kΩ approximately between top and bottom pairs. Therefore, if multiple pressure points are being evaluated simultaneously, no interference will occur between a pair of sensing elements within one film.

Two terminals were connected per electrode to assure the good electrical connection between the terminals and the electrodes, which consisted in aluminium foil glued to the electrodes with *z*-axis conductive adhesive tape (3 M 9703). The maximum terminal-electrode-terminal resistance measured in the prototype was <100 Ω, which warranted a good connection from the terminals to the electrodes.

The developed circuit to measure the sensor response was based on a current source through the sensor, shown in Fig. 8b, which provides a linear voltage drop on the sensor *versus* its resistance, which can be amplified by an instrumentation amplifier (IA) and then fed to the analog-to-digital converter (ADC) of a microcontroller (μC). Finally, the data is sent to a computer *via* a Universal Serial Bus (USB) to universal asynchronous receiver-transmitter (UART) converter.

The selected components of the circuit were as follows: current source: Texas Instruments LM334; IA: Texas Instruments INA321; μC: Nordic Semiconductor nRF52832; USB to UART converter: FTDI Chip FT232X.

The IA is characterized by a gain (*G*) of 5, and amplifies the differential voltage in the sensor to 1.2 V to 2.4 V. The 190 kΩ resistance in the negative pin of the IA simply raises the voltage in this pin to 304 mV, above the minimum operating voltage of the IA. The reference voltage for the ADC comparison was 3.3 V. The sensor was read at a rate of 100 samples per second, using 8 bits, and the UART communication was configured with a baud rate of 1 Mbps.

**Fig. 9:**
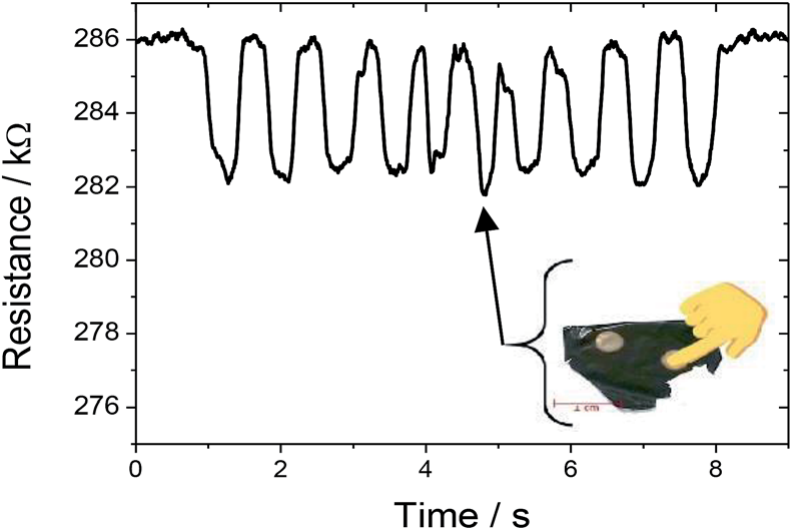
Resistance variation response when force was applied on the sensor by a fingertip touch.

Fig. 9 shows the resistance variation of SF/CNT composites when pressure is applied by a fingertip touch. The resistance response is proportional to the deformation (Fig. 7 and 9) and recovers the initial values when the pressure is released.

Thus, considering environmental issues and the suitable electro-mechanical response of the developed SF/CNT composites, the present work demonstrates the applicability of these natural polymer based composites for the development of the next generation of multifunctional pressure and deformation sensors.

## Conclusions

Silk fibroin/carbon nanotube SF/CNT composites have been prepared for pressure sensor applications. The composites were prepared by the solvent-casting method with different filler contents (0–6 wt%). The CNT fillers were homogeneously distributed in the silk fibroin matrix and the thermal and mechanical properties of these composites are independent of the CNT content.

The addition of CNTs into the silk fibroin matrix affects the β sheet content and the electrical properties (dielectric constant and electrical conductivity). The content of the β sheets decreases and the electrical conductivity increases with increasing CNT content.

The composites show a significant increase of the electrical conductivity with increasing filler content, the percolation threshold being around 3 wt% CNTs.

The piezoresistive behaviour of these composites shows a correspondence with the applied pressure without hysteresis.

In addition, the electro-mechanical behaviour shows good reproducibility over cyclic loading and a suitable pressure sensitivity of ∼4 MPa^−1^ at a small pressures of 0.11 MPa. The results were confirmed by the implementation of a touch/pressure sensor prototype with the corresponding readout electronics.

Thus, it is concluded that the SF/CNT composites show excellent overall performance to contribute to the development of high-performance pressure sensors based on natural polymers.

## Conflicts of interest

There are no conflicts to declare.

## Supplementary Material
